# GDF-15 plasma levels in chronic obstructive pulmonary disease are associated with subclinical coronary artery disease

**DOI:** 10.1186/s12931-017-0521-1

**Published:** 2017-02-28

**Authors:** Carlos H. Martinez, Christine M. Freeman, Joshua D. Nelson, Susan Murray, Xin Wang, Matthew J. Budoff, Mark T. Dransfield, John E. Hokanson, Ella A. Kazerooni, Gregory L. Kinney, Elizabeth A. Regan, J. Michael Wells, Fernando J. Martinez, MeiLan K. Han, Jeffrey L. Curtis, for the COPDGene Investigators

**Affiliations:** 1 Division of Pulmonary & Critical Care Medicine, University of Michigan Health System, 2215 Fuller Road, Ann Arbor, MI 48105-2303 USA; 2 Research Service, VA Ann Arbor Healthcare System, Ann Arbor, MI USA; 3 Department of Biostatistics, School of Public Health, University of Michigan, Ann Arbor, MI USA; 4 Los Angeles Biomedical Research Institute at Harbor-UCLA Medical Center, Torrance, CA USA; 5 The Lung Health Center, Division of Pulmonary, Allergy & Critical Care Medicine, University of Alabama at Birmingham, Birmingham, AL USA; 6 Medical Service, Birmingham Veteran Affairs Medical Center, Birmingham, AL USA; 7 School of Public Health, University of Colorado, Aurora, CO USA; 8 Radiology Department, University of Michigan Health System, Ann Arbor, MI USA; 9 National Jewish Health & Research Center, Denver, CO USA; 10 Pulmonary & Critical Care Medicine Division, Department of Medicine, University of Colorado, Denver, CO USA; 11 Pulmonary & Critical Care Medicine Division, Department of Medicine, Weill Cornell Medical College, New York, NY USA; 12 Graduate Program in Immunology, University of Michigan, 2215 Fuller Road, Ann Arbor, MI 48105-2303 USA; 13 Medical Service, VA Ann Arbor Healthcare System, 2215 Fuller Road, Ann Arbor, MI 48105-2303 USA

**Keywords:** Adult, Biomarkers, Coronary Artery Disease, Cross-Sectional Studies, Multivariate Analysis, Risk Factors

## Abstract

**Background:**

Growth differentiation factor-15 (GDF-15), a cytokine associated with cardiovascular mortality, increases during chronic obstructive pulmonary disease (COPD) exacerbations, but any role in stable COPD is unknown. We tested associations between GDF-15 and subclinical coronary atherosclerosis, assessed by coronary artery calcium (CAC) score, in COPD subjects free of clinical cardiovascular disease (CVD).

**Methods:**

Cross-sectional analysis of COPD participants (GOLD stages 2–4) in the COPDGene cohort without CVD at enrollment, using baseline CAC (from non-EKG-gated chest computed tomography) and plasma GDF-15 (by custom ELISA). We used multinomial logistic modeling of GDF-15 associations with CAC, adjusting for demographics, baseline risk (calculated using the HEART: Personal Heart Early Assessment Risk Tool (Budoff et al. 114:1761-1791, 2006) score), smoking history, measures of airflow obstruction, emphysema and airway disease severity.

**Results:**

Among 694 participants with COPD (47% women, mean age 63.6 years) mean GDF-15 was 1,304 pg/mL, and mean CAC score was 198. Relative to the lower GDF-15 tertile, higher tertiles showed bivariate association with increasing CAC score (mid tertile odds ratio [OR] 1.80, 95% confidence interval [CI] 1.29, 2.51; higher tertile OR 2.86, CI 2.04, 4.02). This association was maintained after additionally adjusting for baseline CVD risk, for co-morbidities and descriptors of COPD severity and impact, markers of cardiac stress (N-terminal pro–B-type natriuretic peptide, troponin T) and of inflammation (Interleukin-6), and in subgroup analysis excluding men, diabetics, current smokers or those with limited ambulation.

**Conclusions:**

In ever-smokers with COPD free of clinical CVD, GDF-15 contributes independently to subclinical coronary atherosclerosis.

**Trial registration:**

ClinicalTrials.gov, NCT00608764. Registered 28 January 2008.

**Electronic supplementary material:**

The online version of this article (doi:10.1186/s12931-017-0521-1) contains supplementary material, which is available to authorized users.

## Background

In chronic obstructive pulmonary disease (COPD), cardiovascular disease (CVD), especially coronary heart disease (CHD), is highly prevalent and impacts mortality and quality of life [1]. COPD is linked to increased risk of overt CVD by substantial epidemiological data, including three large population-based cohorts, two primary care databases, and the nationally representative NHANES survey [1–5]. Uniquely among leading causes of death, worldwide COPD prevalence continues to increase [6, 7], especially among women [8]. Hence, gains in controlling CHD mortality may stall unless the independent contribution from COPD can be combated.

Shared risk factors only partially explain this association between COPD and CHD [9, 10]. Its molecular basis remains undefined, particularly for subclinical disease, the logical window for prevention. One early CHD marker is subclinical coronary artery calcium (CAC), defined as CAC in those free of clinical CVD. CAC is a validated surrogate measure that predicts future cardiovascular events and mortality [11, 12].

Growth-differentiation factor-15 (GDF-15) (gene ID 9518) is a cytokine of the transforming growth factor-β family associated with endothelial and left ventricular dysfunction [13]. Elevated GDF-15 is linked to cardiovascular and all-cause mortality in older adults [14, 15]. We and others recently identified elevated GDF-15 during early COPD exacerbations [16–18].

We hypothesized that in COPD, GDF-15 contributes significantly to subclinical CHD presence and severity as determined by CAC, independently of established CVD risks. We tested this hypothesis using participants with COPD in a well-characterized cohort who were without clinical CVD history and >30 days from exacerbation. Because COPD is heterogeneous [19], with co-morbidity clustering with specific phenotypes [20, 21], we also examined associations with imaging-defined phenotypes.

## Methods

### Study design

This cross-sectional analysis used baseline data from the Genetic Epidemiology of COPD Study (COPDGene®) (ClinicalTrials.gov # NCT00608764), an ongoing multi-center observational cohort designed to identify genetic factors in smoking-related lung disease [22]. COPDGene recruited ever-smokers (≥10 pack-year), non-Hispanic White or African American adults, both sexes, who underwent a detailed phenotypic evaluation, including high-resolution chest computed tomography (HRCT).

### Ethics, consent and permissions

Studies and consent procedures were performed in accordance with the Declaration of Helsinki. Participants understood the study purpose, and provided written informed consent before any procedures. Protocols were approved by Institutional Review Boards at VA Ann Arbor Healthcare System (FWA 00000348) and the University of Michigan (FWA 00004969), approval numbers 2010–120752, HUM000014973, respectively, where we conducted laboratory and statistical analysis, respectively, and at all participating clinical sites.

### Study participants

Participants were chosen from a biomarker pilot project (original *n* = 1000, balanced for sex, age, and equivalent numbers in GOLD stages 2-4), by selecting subjects with available CAC scores and without self-reported CVD. We diagnosed COPD using the fixed-ratio definition [23] (post-bronchodilator forced expiratory volume in the first second over forced vital capacity [FEV1/FVC] ratio <0.7, plus GOLD grade 2–4 obstruction (FEV1% predicted <80%). Spirometry was performed using an EasyOne™ spirometer and NHANES III predictions [24]. Participants were CHD-free on enrollment, based on negative answers to questions about physician diagnosis *(“Have you ever been told by a physician that you have…”)* of heart attack, myocardial infarction, coronary artery disease. We also excluded subjects reporting coronary angioplasty or bypass grafting, congestive heart failure, stroke, transient ischemic attacks or peripheral vascular disease.

### Data collection

#### Outcome

We scored CAC following Agatston’s algorithm [25, 26], from HRCT imaging, un-gated for EKG, and stratified into categories of 0, 1–100, 101–400 and >400 Agatston Units (absence of CAC, mild/minimal, moderate and extensive plaque burden, respectively) [27]. The accuracy and reliability of CAC scoring using un-gated HRCT has been validated in comparisons with EKG-gated studies in COPDGene participants [28].

#### Exposure

We measured GDF-15 levels by ELISA according to manufacturer’s instructions from plasma collected at enrollment and stored at -80 °C until analyzed in duplicate; values are presented in pg/mL; the lower limit of detection is 2 pg/mL.

#### Covariates

Demographics, smoking and medical history were collected using self-administered questionnaires. Co-morbidities and risk factors were ascertained by combining responses to questions on physician-based diagnosis and review of current medications. We considered hypertension, hyperlipidemia and diabetes present when the participant reported either diagnosis or current medication use for that disease. Physical activity was considered limited based on affirming *“Was your walk limited?”* during a 6-min walk test. We calculated 10-year CHD risk based on Personal Heart Early Assessment Risk Tool (HEART score) [25], a validated assessment based on self-reported data [29] suitable for use in subjects without previous CHD diagnosis and in the public domain.

We obtained emphysema percentage and airway wall thickness from volumetric HRCTs obtained at full inflation [22], using 3D Slicer software (www.Slicer.org) and a VIDA Pulmonary Workstation, respectively. Lung areas < -950 Hounsfield Units were considered emphysema, which we stratified as below or ≥10% of lung volume. Airway metrics included the square root of the wall area of a theoretical airway of 10 mm luminal perimeter (Pi10).

### Additional plasma analytes

We measured troponin T, N-terminal-proBNP and IL-6 on the same samples using Luminex assays from Milliplex MAP Human Cardiovascular Disease Panels (EMD Millipore, Billerica, Massachusetts). Assays were run in duplicate according to manufacturer's instructions. The lower limits of detection were 9 pg/mL, 18.8 pg/mL and 0.2 pg/mL, respectively.

### Statistical analysis

Demographics, smoking history, lung function and cardiovascular risk were analyzed by GDF-15 tertiles, using *t*-test or chi-square. We tested association between GDF-15 tertiles and CAC burden categories by two strategies. First, we used regression models with CAC as outcome. Second, we developed sequential multinomial logistic regression models including variables grouped in bloc, starting with GDF tertiles, then adjusting for baseline HEART score, then further adjusting for co-morbidities, measures of lung function, and severity of airway disease and emphysema. We selected logistic modeling as our main approach because it presents outcome and exposure as categories, rather than numeric values, improving their interpretability and clinical relevance. We tested interactions of GDF-15 with imaging characteristics and lung function using similar models. Analyses were performed using Prism 6.0f (GraphPad Software, Inc; La Jolla, CA) and Stata v.12 (College Station, TX).

## Results

**Fig. 1:**
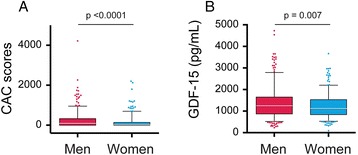
CAC scores & GDF-15 plasma levels by sex. CAC scores were determined by analysis of non-EKG-gated HRCT and GDF-15 plasma levels were measured by ELISA. **a**, CAC scores, as Agatston Units; **b**, GDF-15 concentrations, as pg/mL. Data are median (bar), 25th & 75th percentiles (box), 5th & 95th percentiles (whiskers) with outliers shown as individual points. *p*-values by Mann-Whitney test

We included 694 subjects free of clinical CVD, with large proportions of women and those with chronic bronchitis and >50 pack-years smoking history (Table 1). Almost half were considered at low cardiovascular risk (10 year CHD risk <10%) by HEART score. As a group, men had significantly higher CAC scores (*p* < 0.0001) (Fig. 1a) and GDF-15 levels (*p* = 0.007) (Fig. 1b).

**Table 1:** Characteristics of COPDGene participants with COPD, by GDF-15 tertiles

	All participants (*n* = 694)	Lower (≤966 pg/mL) (*n* = 231)	Mid (967-1438 pg/mL) (*n* = 232)	Higher (≥1439 pg/mL) (*n* = 231)	*p*-value
Sociodemographics					
Age (mean, s.d.)	63.6 (8.4)	59.7 (7.4)	64.6 (8.1)	66.4 (8.3)	<0.001
Females (%)	47.1	49.4	52.6	39.4	0.01
African American (%)	16.3	21.7	13.8	13.4	0.02
BMI (mean, s.d.)	27.3 (6.0)	27.5 (6.0)	26.8 (5.7)	27.6 (6.2)	0.31
Respiratory status					
FEV_1_ in L (mean, s.d.)	1.25 (0.64)	1.28 (0.69)	1.20 (0.61)	1.26 (0.60)	0.30
FEV_1_ % predicted (mean, s.d.)	43.5 (18.8)	42.9 (19.3)	42.9 (18.6)	44.7 (18.4)	0.50
FEV_1_/FVC % (mean, s.d.)	44.4 (13.6)	44.2 (13.8)	43.9 (13.5)	45.4 (13.6)	0.45
GOLD stage (%)					
II	34.1	31.6	33.2	37.7	
III	33.6	32.9	35.8	32.0	
IV	32.3	35.5	31.0	30.3	0.55
mMRC dyspnea score (mean, s.d.)	2.3 (1.4)	2.3 (1.4)	2.2 (1.4)	2.3 (1.4)	0.48
Pack-years smoked (mean, s.d.)	52.2 (25.3)	47.8 (23.6)	52.0 (24.2)	56.8 (27.3)	<0.001
Six-minute walking distance, in f. (mean, s.d.)	1163 (395)	1221 (395)	1180 (377)	1087 (403)	0.001
Chronic bronchitis (%)	27.0	26.4	26.7	27.7	0.94
Emphysema % (mean, s.d.)	17.1 (14.4)	18.2 (14.7)	17.1 (14.2)	16.0 (14.2)	0.26
Pi10 (mean, s.d.)	3.73 (0.14)	3.72 (0.14)	3.71 (0.13)	3.75 (0.14)	0.04
Cardiovascular risk factors (%)					
BMI ≥30	29.0	28.1	26.3	32.5	0.32
Diabetes	8.7	2.6	4.7	18.6	<0.001
Currently smoking	29.8	31.6	25.0	32.9	0.13
Hypertension	43.8	34.6	46.1	50.7	0.002
Hyperlipidemia	36.6	28.1	41.0	40.7	0.005
Aggregate 10-year coronary heart disease risk^a^ (%)					
Low (<10%)	44.5	56.3	47.0	30.3	
Intermediate (10%–20%)	39.9	37.7	38.8	43.3	
High (>20%)	15.6	6.1	14.2	26.4	<0.001
Other co-morbidities (%)					
GERD	28.4	30.3	28.0	26.8	0.70
Asthma	22.5	26.4	22.8	18.2	0.10
Osteoporosis	15.9	13.4	17.2	16.9	0.46
Osteoarthritis	15.0	13.0	13.8	18.2	0.24
Stomach ulcers	8.4	10.4	8.2	6.5	0.31

^a^Based on the HEART score, as described in the Methods section

**Fig. 2:**
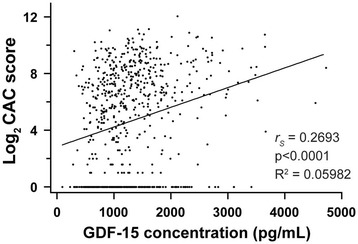
Correlation between Log_2_ CAC & GDF-15 plasma concentrations. *p*-value by Spearman correlation (*r*_*S*_), goodness of fit (R^2^) by linear regression (*n* = 694)

As anticipated, there was a correlation between GDF-15 levels and log_2_CAC that was modest (*r*_*S*_ = 0.2693; R^2^ = 0.05982) but significant (*p* < 0.0001) (Fig. 2). Neither CAC nor GDF-15 correlated with pulmonary artery enlargement, as determined by the ratio of pulmonary artery and aorta diameters (PA:A ratio) [30] (Additional file 1: Table S1), implying that this association did not reflect occult pulmonary hypertension.

Comparing participants by GDF-15 tertiles, those in the higher tertile were significantly older, and more often men and non-Hispanic whites (Table 1). Additionally, high GDF-15 tertile participants had greater smoking histories, more frequently had diabetes, hypertension, hyperlipidemia and obesity, and were more frequently classified at intermediate or high risk by HEART score. However, there were no differences in measures of lung function, dyspnea or emphysema severity (Table 1).

In bivariate and multivariate analyses, GDF-15 levels (in tertiles) were associated with higher CHD risk by HEART score and by its components, including age, smoking and diabetes (Additional file 1: Table S2). Again, however, GDF-15 levels were not influenced by common descriptors of COPD severity (GOLD spirometry severity, FEV1, exacerbation history) or phenotypes (chronic bronchitis, measures of emphysema or airway thickness) (Additional file 1: Table S2).

**Fig. 3:**
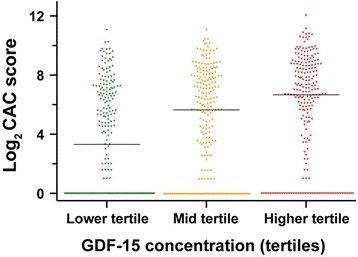
Distribution of Log_2_ CAC by GDF-15 tertiles. Lower tertile (<966 pg/mL) (*n* = 231), mid tertile (967-1438 pg/mL) (*n* = 232), higher tertile (>1439 pg/mL) (*n* = 231)

With increasing GDF-15 tertiles, median CAC scores rose, both as absolute values (not shown) and after logarithmic transformation (Fig. 3). There was a stepped increase in CAC scores with increasing GDF-15: relative to the lower GDF-15 tertile, we found 1.19-fold and 2.10-fold increases, respectively, in mid- and highest tertiles. Nevertheless, even in the highest tertile, not all subjects had measurable CAC (Fig. 3).

In logistic models, GDF-15 contributed significantly to CAC burden (Table 2). The association of GDF-15 and CAC (by severity groups) was strong in the bivariate model (Model 1), and remained of similar magnitude after additionally adjusting for baseline cardiovascular risk (Model 2), for co-morbidities and measures of lung function (Model 3), and for biomarkers (Model 4). Importantly, the magnitude of the GDF-15-CAC association was at least as great as that of the combined classical risk factors included in the HEART score.

**Table 2:** Adjusted associations of GDF-15 levels and of cardiovascular risk with CAC score among COPDGene participants with COPD (*n* = 694)

	Model 1^a^	Model 2^b^	Model 3^c^	Model 4^d^
GDF-15 level (in tertiles)				
Lower tertile	Ref.	Ref.	Ref.	Ref.
Intermediate tertile	2.52 (1.10, 2.71)	2.30 (1.19, 4.44)	2.16 (1.11, 4.20)	1.87 (0.88, 3.99)
Higher tertile	5.70 (3.08, 10.56)	4.53 (2.40, 8.54)	4.22 (2.20, 8.07)	4.28 (2.09, 8.76)
Coronary heart disease risk group^e^				
Low (<10%)		Ref.	Ref.	Ref.
Intermediate (10%-20%)		1.49 (0.86, 2.57)	1.46 (0.83, 2.58)	1.33 (0.69, 2.55)
High (>20%)		2.89 (1.45, 5.77)	3.77 (1.79, 7.94)	3.97 (1.68, 9.35)

All entries represent risk ratio of being in the higher group of CAC and its 95% CI, based on multinomial logistic regression models, in which GDF-15 tertiles are compared again the lower tertile as reference, and the groups of cardiovascular risk by the HEART score are compared with the low-risk group as reference. ^a^Model 1: Bivariate. ^b^Model 2: Model 1 additionally adjusted for baseline coronary heart disease risk group. ^c^Model 3: Model 2 additionally adjusted for race, GOLD spirometry stage, pack-years smoked, chronic bronchitis symptoms, history of exacerbations, measures of emphysema and airway thickness, and co-morbidities (asthma, GERD, mobility-related diseases). ^d^Model 4: Model 3 additionally adjusted for N-terminal pro–B-type natriuretic peptide, troponin T and Interleukin-6. ^e^Based on the HEART score, as described in the Methods section

Next, we performed a sensitivity analysis, testing the association in subgroups chosen to minimize known correlation with CAC. First, we restricted analysis to women (Table 3). Second, we excluded those with diabetes, a major contributor to the HEART score that is associated with both CAC and GDF-15 elevation. Third, we restricted analysis to those not smoking at enrollment. Fourth, because low physical activity and a sedentary lifestyle are strong CVD risk factors, we repeated the analysis using only those reporting no walking limitations. None of these pre-planned subgroups analysis significantly modified the association of GDF-15 (higher tertile) with CAC (Table 3).

**Table 3:** Multivariate models of association of GDF-15 with CAC score among different subgroups of COPDGene participants with COPD

	Restricted to			
	Female participants (*n* = 327)	Participants without diabetes (*n* = 634)	Participants not currently smoking (*n* = 487)	Participants with no walking limitation (*n* = 304)
GDF-15 level (in tertiles)				
Lower tertile	Ref.	Ref.	Ref.	Ref.
Intermediate tertile	2.36 (0.84, 6.64)	1.95 (0.99, 3.84)	1.40 (0.66, 2.98)	1.48 (0.57, 3.87)
Higher tertile	4.56 (1.62, 12.8)	4.00 (2.06, 7.78)	2.41 (1.13, 5.12)	3.76 (1.63, 10.4)

All entries represent risk ratio of being in the higher group of CAC and its 95% CI, based on multinomial logistic regression models

All models additionally adjusted for race, GOLD spirometry stage, pack-years smoked, chronic bronchitis symptoms, history of exacerbations, and co-morbidities (asthma, GERD, mobility-related diseases)

Finally, we tested whether different markers of COPD phenotype and severity modified this association (Additional file 1: Table S3). Given correlations between airway thickness and cardiometabolic diseases [21], we used two approaches, restricting the analysis to those with less airway disease (lower two tertiles of pi10 distribution) or those with greater emphysema. We also restricted analysis to subjects with less severe airflow obstruction (defined either as spirometry stage 2–3 or FEV_1_ ≥ 1 L). None of these analyses (Additional file 1: Table S3) changed the associations between GDF-15 and CAC.

## Discussion

This analysis of 694 ever-smokers without clinical CVD at enrollment to the COPDGene cohort demonstrates that GDF-15 independently contributes to subclinical atherosclerosis in COPD. Although GDF-15 is elevated in pulmonary hypertension of any cause, we found no correlation with PA:A ratio. The association of GDF-15 with CAC showed a gradient, and both its magnitude, and its persistence in subgroup analyses, imply that GDF-15 is not simply a surrogate for traditional risk factors. Instead, our findings support the concept [31] that adding GDF-15 to established indicators improves CVD risk estimation. This supposition, which will require additional testing, is most relevant to younger COPD subjects in low GOLD stages, in whom long-term CVD risk could exceed that of respiratory death. Thus, these data have important implications for reducing the impact of multi-morbidity in ever-smokers and also provide potential mechanistic insight into the link between COPD and overt CVD [1–5].

Reduced lung function as powerfully predicts CVD mortality as total cholesterol [32] but has never been embraced as a modifiable risk factor. Our findings are consistent with reported associations between smoking, airflow limitation and subclinical atherosclerosis, as measured by carotid intimal thickening [33]. We extend those results by using CAC score, which has higher predictive value for incident CVD than carotid plaque [34], and by showing independence from active smoking. That point is important, as GDF-15 is elevated by smoking [3] and cigarette smoke induces its production by human lung epithelial cells in vitro [35].

We extend to stable COPD without clinical CVD the evidence that GDF-15 is an informative biomarker, as established in overt CVD [36, 37]. To our knowledge, the only previous study that analyzed GDF-15 in stable COPD [38] found elevated levels relative to healthy never-smokers, but was underpowered (*n* = 15) to examine other correlations. Our findings agree with two large (combined *n* = 2395) studies of community-dwelling elderly individuals without CVD history in showing that GDF-15 adds significant value to predictive models based on accepted CVD biomarkers [14, 15]. Those longitudinal studies additionally found that baseline GDF-15 levels independently predicted all-cause and cardiovascular mortality [14, 39], which we hope to examine in our 694 COPDGene participants. Importantly, associations of GDF-15 with mortality in the general population equal or exceed that of N-terminal pro-BNP and C-reactive protein [14, 15].

However, the clinical value of CAC is debated, and currently only recommended for selected asymptomatic adults at intermediate risk [40]. Data on CAC in COPD are conflicting. A single-center case-control study (*n* = 162) found no difference in CAC despite higher major adverse cardiovascular events in COPD [41]. By contrast, CAC on non-EKG-gated CTs correlated significantly with all-cause mortality in ECLIPSE, in an analysis including those with known CVD [27]. Interestingly, CAC scores in ECLIPSE did not correlate with FEV_1_ or exacerbation history. Similarly, we did not find a correlation of baseline GDF-15 levels and common markers of COPD severity and phenotypes. Thus, GDF-15 appears to be a prognostic biomarker of CVD co-morbidity, but not a predictive biomarker of COPD outcomes, in agreement with recent findings suggesting that endothelial dysfunction predicts atherosclerosis in COPD, but does not contribute to airflow limitation [42].

As a biomarker, GDF-15 has both strengths and limitations. Although GDF-15 appears to reflect an integrated stress response [15, 37], understanding the significance of an elevated concentration is complicated by its production by multiple cell-types and in response to disparate stimuli [43]. Elevated GDF-15 may reflect endothelial cell dysfunction, common in advanced COPD and proposed as a shared pathway in CVD development [44]. However, endothelial dysfunction in COPD appears to relate in part to intrathoracic mechanics rather than inflammation [45], and may associate with specific COPD phenotypes [46]. Hence, GDF-15 might be elevated in individual COPD patients for diverse reasons. Thus, like TGF-β itself, GDF-15 may not be simply good or bad, but either, depending on context, underlining the need for greater understanding of its signal transduction in specific cell-types and clinical settings.

Despite the strengths of a well-characterized cohort in which the CAC outcome has been validated [28], potential limitations include information bias, as coexistent diseases were self-reported. Although a significant concern for incident diseases, information bias is less so for prevalent diseases [47]. We attempted to improve the accuracy of excluding baseline CVD by supplementing self-reported diagnoses with history of related treatment or interventions. A second potential limitation is our use of a self-reported risk score to calculate future CHD likelihood, instead of biologic measures included in some common predictive instruments. However, the HEART score is a validated instrument specifically designed to use self-reported data in epidemiologic studies [29]. Finally, our cross-sectional design limits inferences about temporal associations. Nevertheless, we consider our findings robust, consistent with current understanding of associations between CHD and COPD and an advance in understanding the mechanisms involved in both diseases.

## Conclusions

We demonstrate that in COPD subjects free of clinical CVD, subclinical atherosclerosis (measured as CAC on ungated HRCT) is frequent; and that GDF-15 contributes, independently of common cardiovascular risk factors and measures of COPD severity or phenotype, to its presence and severity.

## Additional file

Additional file 1: Tables S1-S3. ﻿**Table S1﻿﻿.﻿** ﻿Lack of association of GDF-15 levels or CAC score with PA:A ratio*. **Table S2.** Bivariate and Multivariate Associations with GDF-15 among COPDGene participants with COPD (*n* = 694). **Table S3.** Multivariate models of association of GDF-15 with CAC score among different subgroups of COPDGene participants with COPD. (DOCX 33 kb)

## Abbreviations

CAC: Coronary artery calcification
CHD: Coronary heart disease
CI: Confidence interval
COPD: Chronic obstructive pulmonary disease
COPDGene: Genetic Epidemiology of COPD Study
CVD: Cardiovascular disease
GDF-15: Growth differentiation factor-15
GOLD: Global Initiative for Obstructive lung Disease
HEART: Personal heart early assessment risk tool
HRCT: High-resolution chest computed tomography
mMRC: Modified medical research council
NHANES: National health and nutrition examination survey
NT-proBNP: N-terminal pro–B-type natriuretic peptide
Pi10: The square root of the wall area of a theoretical airway of 10 mm luminal perimeter
