# Supplementary material for: GDF-15 plasma levels in chronic obstructive pulmonary disease are associated with subclinical coronary artery disease
Source: Respir Res. 2017 Feb 28;18:42. doi: 10.1186/s12931-017-0521-1 (PMC5331711; doi:10.1186/s12931-017-0521-1)
Supplement: Additional file 1: Tables S1-S3. — ﻿Table S1﻿﻿.﻿ ﻿Lack of association of GDF-15 levels or CAC score with PA:A ratio*. Table S2. Bivariate and Multivariate Associations with GDF-15 among COPDGene participants with COPD (n = 694). Table S3. Multivariate models of association of GDF-15 with CAC score among different subgroups of COPDGene participants with COPD. (DOCX 33 kb) [file 12931_2017_521_MOESM1_ESM.docx]

**SUPPLEMENTAL MATERIALS**

**GDF-15 plasma levels in chronic obstructive pulmonary disease are associated
with subclinical coronary artery disease**

Carlos H. Martinez, M.D., M.P.H. ^1^, Christine M. Freeman, Ph.D. ^1,2^, Joshua D. Nelson, B.S. ^1^, Susan Murray, ScD ^3^, Xin Wang, Ph.D. ^3^, Matthew J. Budoff, M.D. ^4^,
Mark T. Dransfield, M.D. ^5, 6^, John E. Hokanson, Ph.D. ^7^, Ella A. Kazerooni, M.D., M.S. ^8^
Gregory L. Kinney, Ph.D. ^7^, Elizabeth A. Regan, M.D., Ph.D. ^9, 10^, J. Michael Wells, M.D. ^5, 6^, Fernando J. Martinez, M.D., M.S. ^11^, MeiLan K. Han, M.D., M.S. ^1^,
Jeffrey L. Curtis, M.D. ^1, 12, 13^ for the COPDGene Investigators.

^1^ Division of Pulmonary & Critical Care Medicine, University of Michigan Health System, Ann Arbor, MI

^2^ Research Service, VA Ann Arbor Healthcare System, Ann Arbor, MI

^3^ Department of Biostatistics, School of Public Health, University of Michigan, Ann Arbor, MI

^4^ Los Angeles Biomedical Research Institute at Harbor-UCLA Medical Center, Torrance, CA

^5^ The Lung Health Center, Division of Pulmonary, Allergy & Critical Care Medicine, University of Alabama at Birmingham, Birmingham, AL

^6^ Medical Service, Birmingham Veteran Affairs Medical Center, Birmingham, AL

^7^ School of Public Health, University of Colorado, Aurora, CO

^8^ Radiology Department, University of Michigan Health System, Ann Arbor, MI

^9^ National Jewish Health & Research Center, Denver, CO

^10^ Pulmonary & Critical Care Medicine Division, Department of Medicine, University of Colorado, Denver, CO

^11^ Pulmonary & Critical Care Medicine Division, Department of Medicine, Weill Cornell Medical College, New York, NY

^12^ Graduate Program in Immunology, University of Michigan, Ann Arbor, MI

^13^ Medical Service, VA Ann Arbor Healthcare System, Ann Arbor, MI

**Corresponding author**:

Jeffrey L. Curtis, M.D.

Pulmonary & Critical Care Medicine Section (506/111G)

VA Ann Arbor Healthcare System
2215 Fuller Road; Ann Arbor, MI 48105-2303, U.S.A.

E-mail: [jlcurtis@umich.edu](mailto:jlcurtis@umich.edu)

**Additional file 1: Table S1.**  Lack of association of GDF-15 levels or CAC score with PA:A ratio*.

|  | Risk Ratio | 95% CI | p-value |
| --- | --- | --- | --- |
| GDF-15 to PA:A ratio |  |  |  |
| Ratio (by one unit increment) | 0.28 | 0.07, 1.09 | 0.06 |
| Ratio ≥1 (versus <1) | 0.87 | 0.57, 1.33 | 0.52 |
| CAC to PA:A ratio |  |  |  |
| Ratio (by one unit increment) | 0.27 | 0.05, 1.51 | 0.13 |
| Ratio ≥1 (versus <1) | 0.71 | 0.42, 1.21 | 0.20 |

*, PA:A, ratio of the diameter of the pulmonary artery to diameter of the aorta as measured on CT scan [^1^](#_ENREF_1).

**Additional file 1: Table S2**. Bivariate and Multivariate Associations with GDF-15 among COPDGene participants with COPD (n=694).

|  | Bivariate | Multivariate* |
| --- | --- | --- |
| By 10-year coronary heart disease risk group^&^ |  |  |
| Low (<10%) | Ref. | Ref. |
| Intermediate (10%-20%) | 1.74 (1.28, 2.35) | 1.57 (1.15, 2.15) |
| High (>20%) | 4.37 (2.85, 6.68) | 4.23 (2.70, 6.63) |
| Sociodemographics |  |  |
| Age (per year increment) | 1.08 (1.06, 1.10) | 1.09 (1.07, 1.12) |
| Females (Reference is Male) | 0.74 (0.56, 0.98) | 0.80 (0.59, 1.09) |
| African American (Reference is Non-Hispanic White) | 0.63 (0.43, 0.91) | 0.75 (0.48, 1.15) |
| Respiratory status |  |  |
| Spirometry GOLD stage IV  (Reference is GOLD II-III) | 0.84 (0.62, 1.12) | 0.92 (0.65, 1.31) |
| Pack-years smoked (per 10 pack-year increment) | 1.11 (1.05, 1.18) | 1.03 (0.97, 1.10) |
| ≥2 exacerbation per annum (Reference 0-1) | 0.85 (0.62, 1.17) | 1.04 (0.73, 1.50) |
| Chronic bronchitis present (Reference is absent) | 1.05 (0.77, 1.43) | 0.97 (0.68, 1.37) |
| Emphysema >10% | 0.81 (0.61, 1.07) | 0.88 (0.62, 1.24) |
| Higher tertile of airway thickness | 1.34 (0.93, 1.93) | 1.33 (0.89, 1.97) |
| Other co-morbidities (%) |  |  |
| Asthma | 0.70 (0.51, 0.97) | 0.92 (0.64, 1.32) |
| GERD | 0.88 (0.65, 1.19) | 0.89 (0.64, 1.24) |
| Mobility-related diseases | 1.14 (0.85, 1.52) | 1.01 (0.74, 1.39) |
| Individual cardiovascular risk factors^£^ |  |  |
| Obesity (BMI ≥30) | 1.17 (0.87, 1.59) | 1.02 (0.73, 1.45) |
| Diabetes | 5.85 (3.28, 10.43) | 5.49 (2.95, 10.22) |
| Currently smoking | 1.05 (0.78, 1.42) | 1.94 (1.31, 2.89) |
| Hypertension | 1.63 (1.24, 2.15) | 1.24 (0.89, 1.66) |
| Hyperlipidemia | 1.50 (1.13, 1.99) | 1.04 (0.75, 1.43) |

All entries represent OR and its 95% CI, based on ordinal logistic regression.

*Multivariate model includes all variables listed in the table, unless otherwise explained.

^&^Based on the HEART score, as described in the Methods section.

^£^From a separate model replacing HEART score by its individual components.

**Additional file 1: Table S3**. Multivariate models of association of GDF-15 with CAC score among different subgroups of COPDGene participants with COPD.

|  | Restricted to participants with | | | |
| --- | --- | --- | --- | --- |
|  | Less airway disease  (n=567) | Emphysema >10%  (n=389) | GOLD 2-3 spirometry  (n=389) | FEV_1_ ≥1.0 L  (n=398) |
| GDF-15 level (in tertiles) | |  |  |  |
| Lower tertile | Ref. | Ref. | Ref. | Ref. |
| Intermediate tertile | 1.64 (1.14, 2.38) | 1.85 (1.19, 2.88) | 1.42 (0.61, 3.34) | 1.22 (0.49, 3.07) |
| Higher tertile | 2.59 (1.73, 3.88) | 2.95 (1.83, 4.77) | 3.09 (1.38, 6.95) | 2.60 (1.08, 6.25) |

All entries represent OR and its 95% CI, based on ordinal multinomial logistic regression models.

All models additionally adjusted for race, GOLD spirometry stage, pack-years smoked, chronic bronchitis symptoms, history of exacerbations, and co-morbidities (asthma, GERD, mobility-related diseases).

# Reference

1 Wells JM, Washko GR, Han MK, et al. Pulmonary arterial enlargement and acute exacerbations of COPD. *N Engl J Med* 2012; 367:913-921.
